# Transcriptomic analysis of the liver of cholesterol-fed rabbits reveals altered hepatic lipid metabolism and inflammatory response

**DOI:** 10.1038/s41598-018-24813-1

**Published:** 2018-04-24

**Authors:** Weirong Wang, Yulong Chen, Liang Bai, Sihai Zhao, Rong Wang, Baoning Liu, Yali Zhang, Jianglin Fan, Enqi Liu

**Affiliations:** 1 Research Institute of Atherosclerotic Disease, Xi’an Jiaotong University Cardiovascular Research Center, Xi’an, Shaanxi 710061 China; 2 Laboratory Animal Center, Xi’an Jiaotong University Health Science Center, Xi’an, Shaanxi 710061 China; 3 Shaanxi Key Laboratory of Ischemic Cardiovascular Disease, Institute of Basic and Translational Medicine, Xi’an Medical University, Xi’an, Shaanxi 710021 China; 4 Department of Molecular Pathology, Interdisciplinary Graduate School of Medicine and Engineering, University of Yamanashi, Yamanashi, 409–3898 Japan

## Abstract

Rabbits are a suitable animal model for atherosclerosis due to their sensitivity to dietary cholesterol. Moreover, rabbits have lipoprotein profiles that are more similar to humans than those of other laboratory animals. However, little is known about the transcriptomic information related to atherosclerosis in rabbits. We aimed to determine the changes in the livers of rabbits fed a normal chow diet (control) or high cholesterol diet (HCD) by histological examinations and RNA sequencing analysis. Compared with the control group, the lipid levels and small LDL subfractions in plasma were increased, and aortic atherosclerotic plaques were formed in the HCD group. Most importantly, HCD resulted in lipid accumulation and inflammation in the livers. Transcriptomic analysis of the liver showed that HCD induces 1183 differentially expressed genes (DEGs) that mainly participate in the regulation of inflammation and lipid metabolism. Furthermore, the signaling pathways involved in inflammation and lipid metabolism were enriched by KEGG pathway analysis. In addition, hepatic DEGs of the HCD group were further validated by real-time PCR. These results suggest that HCD causes liver lipid accumulation and inflammatory response. Although the relationships between these hepatic changes and atherogenesis need further investigation, these findings provide a fundamental framework for future research on human atherosclerosis using rabbit models.

## Introduction

Atherosclerosis is a complex multifactorial disease of the aorta and muscular arteries and the leading cause of morbidity and mortality throughout the world^1^. Many laboratory animal models have been used to study the process of atherogenesis, including mice, rabbits, pigs and nonhuman primates. In particular, mouse models are widely used due to the relatively short time frame for the progression of atherosclerosis and the relative ease of genetic manipulation^2^. While not all aspects of mouse atherosclerosis are identical to humans, several characteristics of lipid metabolism in rabbits make them particularly suitable for the study of human hypercholesterolemia and atherosclerosis. First, a large body of evidence suggests that high levels of plasma cholesterol, especially low-density lipoprotein (LDL)-cholesterol, result in atherosclerotic lesion formation in humans^3^. Unlike mice, in which high-density lipoproteins (HDLs) are the predominant plasma lipoproteins, rabbit lipoprotein profiles are LDL-rich, similar to those of humans^4,5^. Second, it has been reported that cholesteryl ester transfer protein (CETP) plays a central role in lipid metabolism and atherosclerotic processes^6^. Rabbits have abundant CETP activity in the plasma as do humans, whereas mice do not have an endogenous CETP gene^7,8^. Third, rabbits do not have hepatic ApoB mRNA editing activity as humans, so rabbit ApoB-48 is only present in chylomicrons as in humans. However, ApoB-48 is present in all ApoB-containing particles such as very low-density lipoproteins (VLDLs), LDLs and chylomicrons in mice^9^. In addition, hepatic lipase (HL), which plays a major role in lipoprotein metabolism, is bound to the vascular endothelium in rabbits and humans, whereas the majority of HL is circulating in the plasma in mice^10,11^. Therefore, rabbits provide a unique system to study the initiation and progression of atherosclerosis in humans. Despite the importance of rabbit models for the study of human hypercholesterolemia and atherosclerosis, little is known about the transcriptomic information related to atherosclerosis in rabbits.

Next-generation sequencing platforms have made genomic and transcriptomic analyses affordable for researchers to elucidate the molecular mechanisms of human diseases. RNA sequencing (RNA-Seq) uses massively parallel sequencing to analyze the transcriptome at higher resolution than Sanger sequencing- and microarray-based methods^12,13^. We envision that investigation of the transcriptomics in rabbits fed a high cholesterol diet can provide a better understanding of the biochemical and molecular processes involved in the initiation and progression of atherosclerosis in this model.

Therefore, in the present study, we conducted RNA-Seq analysis of livers in rabbits fed a high cholesterol diet coupled with analyses of the plasma lipids and lipoprotein subfractions, aortic atherosclerotic lesions and liver pathology.

## Results and Discussion

### Plasma lipid levels and lipoprotein subfractions

**Figure 1:**
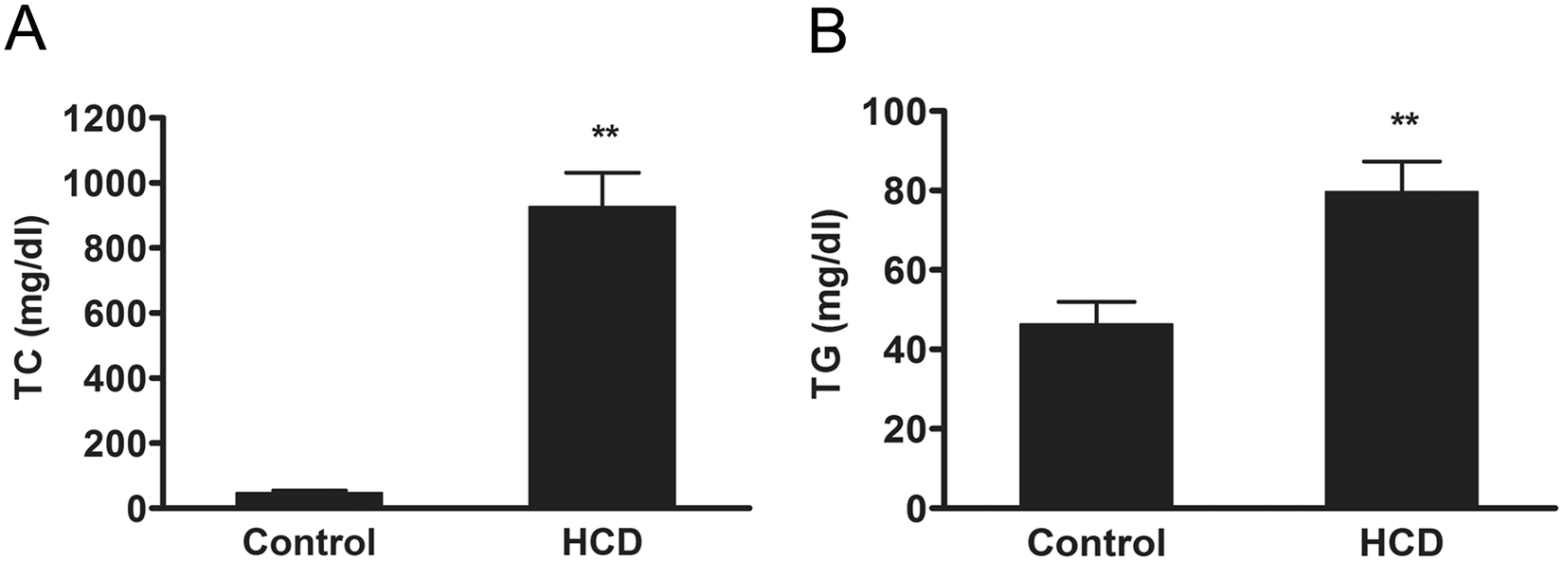
The plasma lipid levels in rabbits. The rabbits were fed a normal chow diet or 0.3% high cholesterol diet for 16 weeks. The plasma levels of TC and TG were measured. Data are expressed as the mean ± SEM. n = 10 for each group. **P < 0.01 *vs*. the control group.

Compared with the normal chow diet (control) group, the plasma levels of total cholesterol (TC) and triglycerides (TG) were increased by 19.4-fold and 1.7-fold in high cholesterol diet (HCD)-fed rabbits for 16 weeks, respectively (Fig. 1, *P* < 0.01).

**Figure 2:**
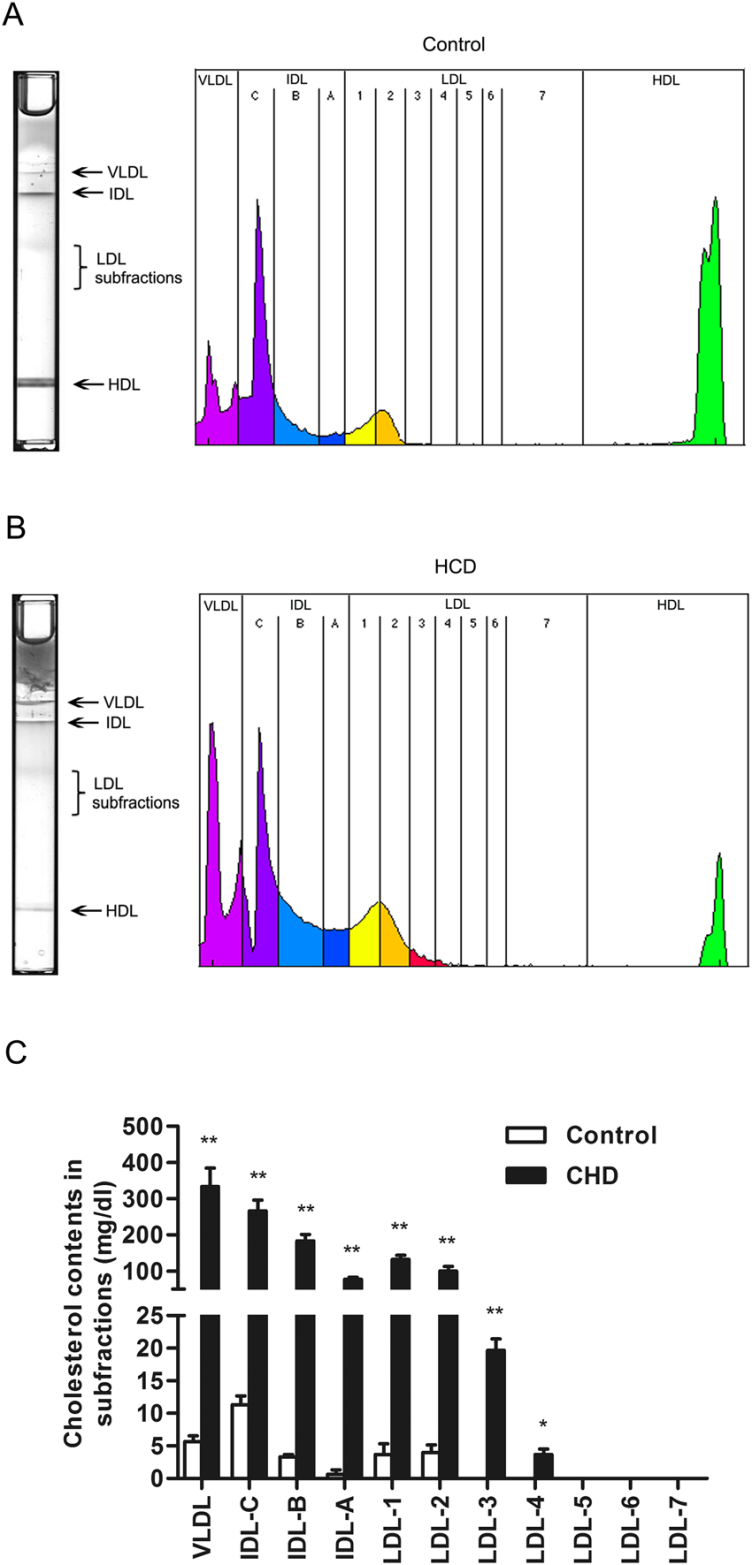
The plasma lipoprotein subfraction analysis in rabbits. Blood samples in the control group and HCD group were used for lipoprotein subfraction analysis. Data are expressed as the mean ± SEM. n = 3 for each group. *P < 0.05 and **P < 0.01 *vs*. the control group.

As shown in Fig. 2, plasma lipoprotein subfraction analysis showed that the levels of VLDLs, intermediate-density lipoproteins (IDLs) and LDLs were markedly increased in the HCD group compared with the control group. It is well established that increased LDL levels are considered a major risk factor for atherosclerosis^14,15^. However, an increasing number of studies have found that the disproportionate number of small and denser LDL particles is an independent risk factor for cardiovascular diseases because small LDL particles may reside longer in circulation and may be more prone to uptake by macrophages in atherosclerosis^16,17^. Therefore, we further analyzed the size distribution of LDL particles using the Lipoprint LDL System. The system can resolve plasma lipoproteins to discrete bands consisting of VLDL; IDL bands A, B, and C; LDL subfractions 1 to 7. Subfraction 1 represents large LDL particles, subfraction 2 indicates intermediate LDL particles, and subfractions 3–7 refer to small LDL particles^18^. We found that small LDL particles were basically not present in the control rabbits whereas they (fractions 3–4) were dramatically increased in the HCD-fed rabbits (Fig. 2C). It has been reported that small LDL particles are more atherogenic than large buoyant LDL particles^19,20^. Recent clinical trials also showed that small LDL particles were markers of early atherosclerosis, and the measurement of small LDL particles provided important information in the risk assessment for atherosclerotic disease^21,22^. In addition, there were obvious differences in the plasma levels of VLDL, IDL-C, IDL-B and IDL-A between the HCD group and the control group in our study (*P* < 0.01). Data from the present study showed that high dietary cholesterol increased the plasma levels of TC and TG, and increased plasma TC levels were mainly caused by elevated small LDL particles in rabbits.

### Gross and histological changes in atherosclerotic lesions

**Figure 3:**
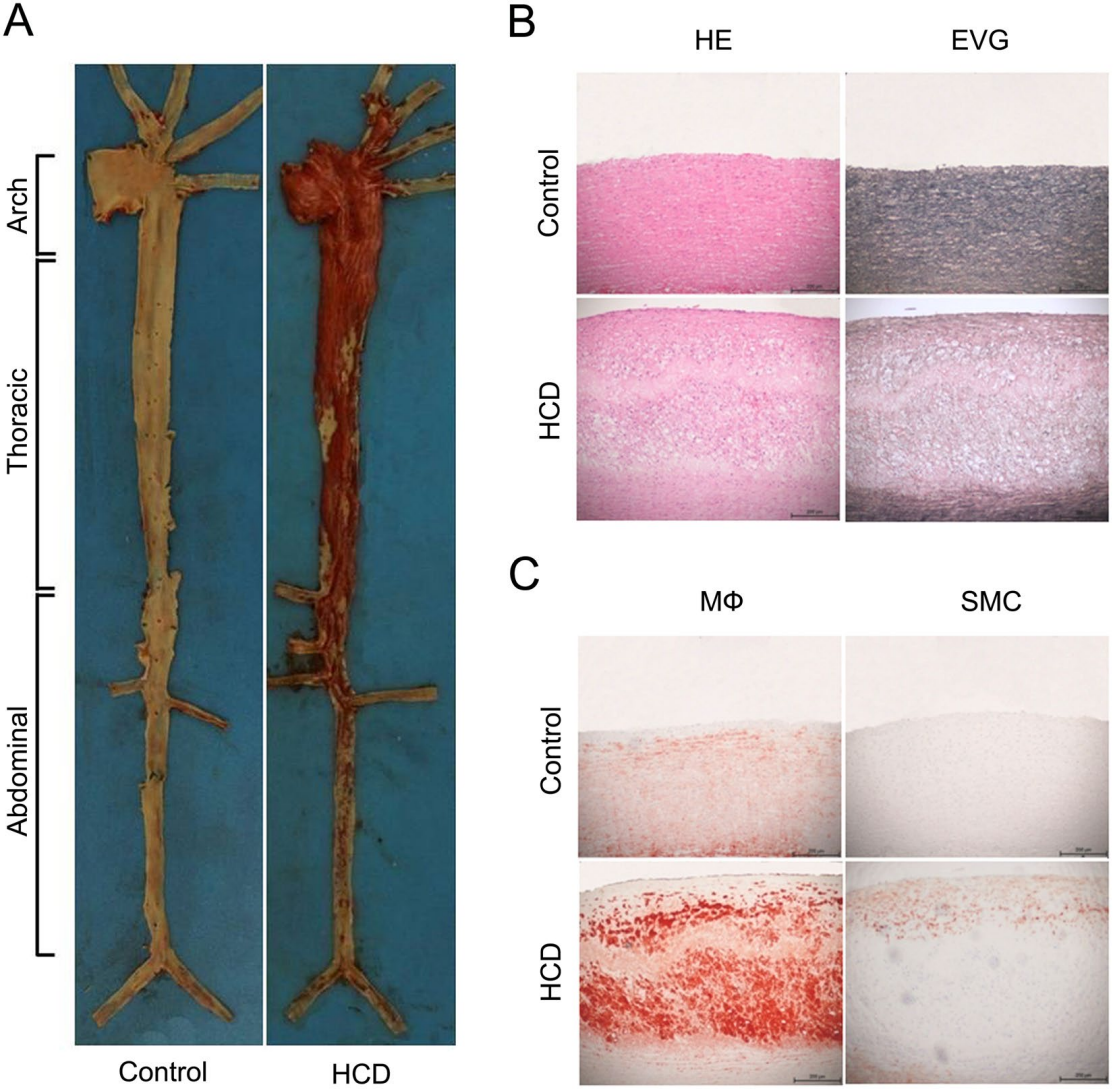
Gross and histological changes in atherosclerotic lesions in rabbits. (**A**) Representative pictures of Sudan IV staining in aortas. (**B**) The aortic atherosclerotic lesions were stained with H&E or EVG. (**C**) The aortic atherosclerotic lesions were stained with antibodies against MФ or SMC by immunohistochemistry. Bar = 200 μm.

Gross atherosclerotic lesions and histological features in the aorta were examined. Aortas of rabbits were collected and stained with Sudan IV. Compared with the control group, which contained no visible lesions, *en face* lesions in the HCD group were remarkable and distributed from the aortic arch, thoracic aorta to abdominal aorta with predominance in the aortic arch (Fig. 3A).

To further analyze the histological changes of atherosclerotic lesions, we examined the intimal lesions in the aortic arch using hematoxylin and eosin (H&E) and Elastica van Gieson (EVG) staining. We found that the intimal lesions were mainly composed of macrophage-derived foam cells, whereas no lesions were found in the control group (Fig. 3B). It is generally accepted that the enhancement of intimal macrophage (MФ) accumulation may increase the vulnerability of the plaques or make the plaques prone to rupture, leading to thrombosis in atherosclerosis^23^. In addition, vascular smooth muscle cell (SMC) proliferation also plays a crucial role in the progression of atherosclerosis^24^. Immunohistochemical staining showed that in addition to macrophages, the lesions also contained many SMCs (Fig. 3C). Thus, aortic atherosclerotic lesions were well formed in rabbits fed a 0.3% cholesterol diet for 16 weeks.

### Histological changes in liver

**Figure 4:**
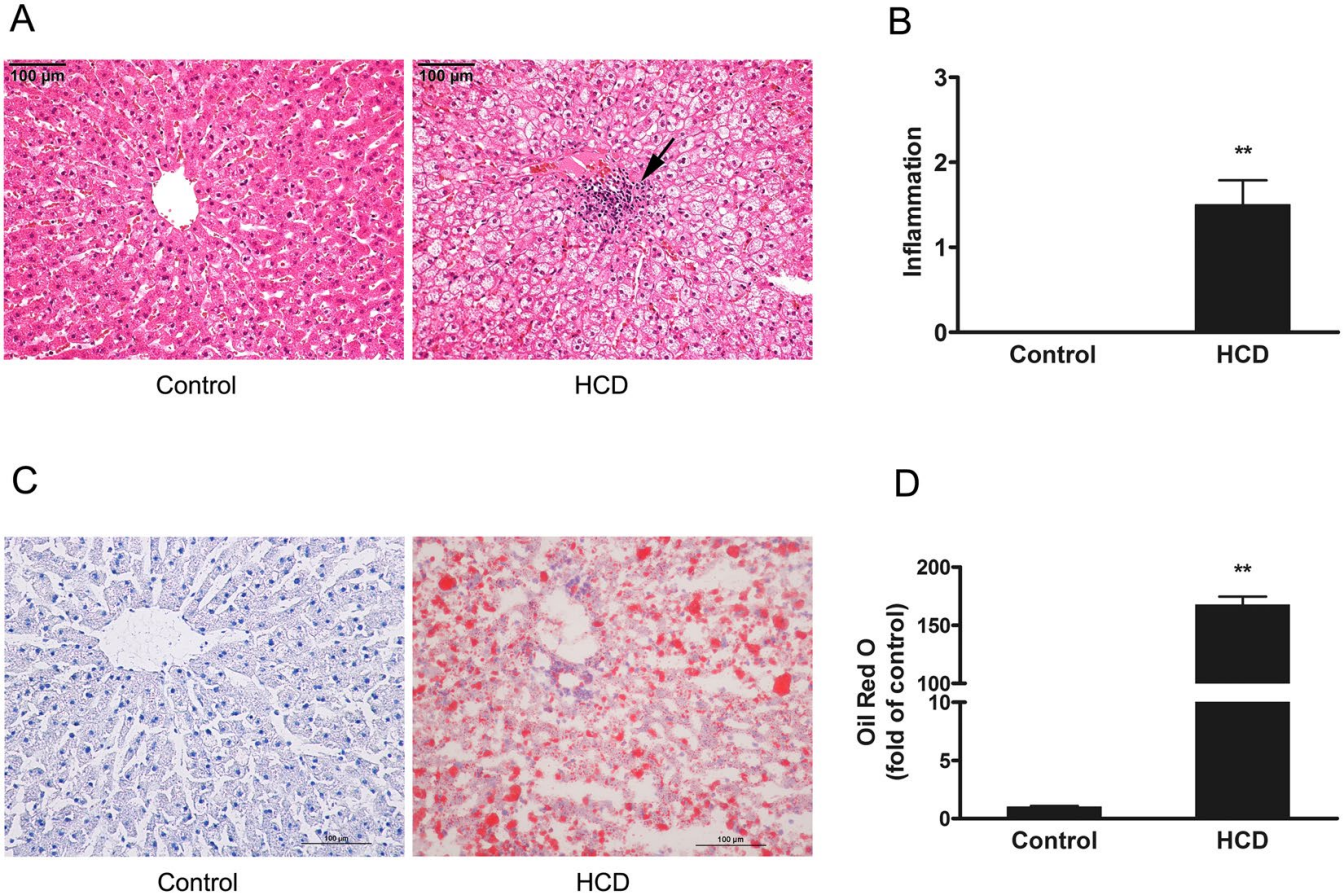
Histological changes in the livers of rabbits by H&E and Oil Red O staining. (**A**) Compared to the control liver, the hepatocytes of HCD-fed rabbits show fatty degeneration and accumulation of mononuclear infiltration in the center (indicated by an arrow). (**B**) Hepatic inflammation was quantified using the Brunt classification. (**C**) Representative Oil Red O staining of livers in the control group and HCD group. (**D**) Oil Red O staining area was measured using the WinROOF image analysis software. Data are expressed as the mean ± SEM. n = 4 for each group. **P < 0.01 *vs*. the control group. Bar = 100 μm.

Accumulating evidence suggests that the liver plays a key role in the inflammatory response evoked by dietary cholesterol. Clinical studies found that liver-derived inflammation markers such as C-reactive protein and serum amyloid A (SAA) were rapidly increased after consumption of an excess amount of dietary cholesterol, thereby promoting the onset of early aortic lesion formation^25,26^. These results indicated that liver plays a key role in the inflammatory response evoked by dietary constituents. We found that there were significant steatosis, ballooning and inflammation in the HCD group compared with the control group (Fig. 4A). In addition, histological scoring for inflammation was based on the Brunt classification^27,28^. There was a trend toward higher inflammation in the HCD group compared with the control group (*P* < 0.01) (Fig. 4B). The results of Oil Red O revealed clear lipid accumulation and severe macrovesicular hepatic steatosis in the HCD group compared with the control group (Fig. 4C and D). These results suggest that HCD could cause fatty degeneration and inflammatory response in the liver. In support of our observations, it has been shown that dietary cholesterol induced pro-inflammatory gene expression in the livers of ApoE*3Leiden transgenic mice^29^.

### RNA-Seq analysis of hepatic gene expression

**Figure 5:**
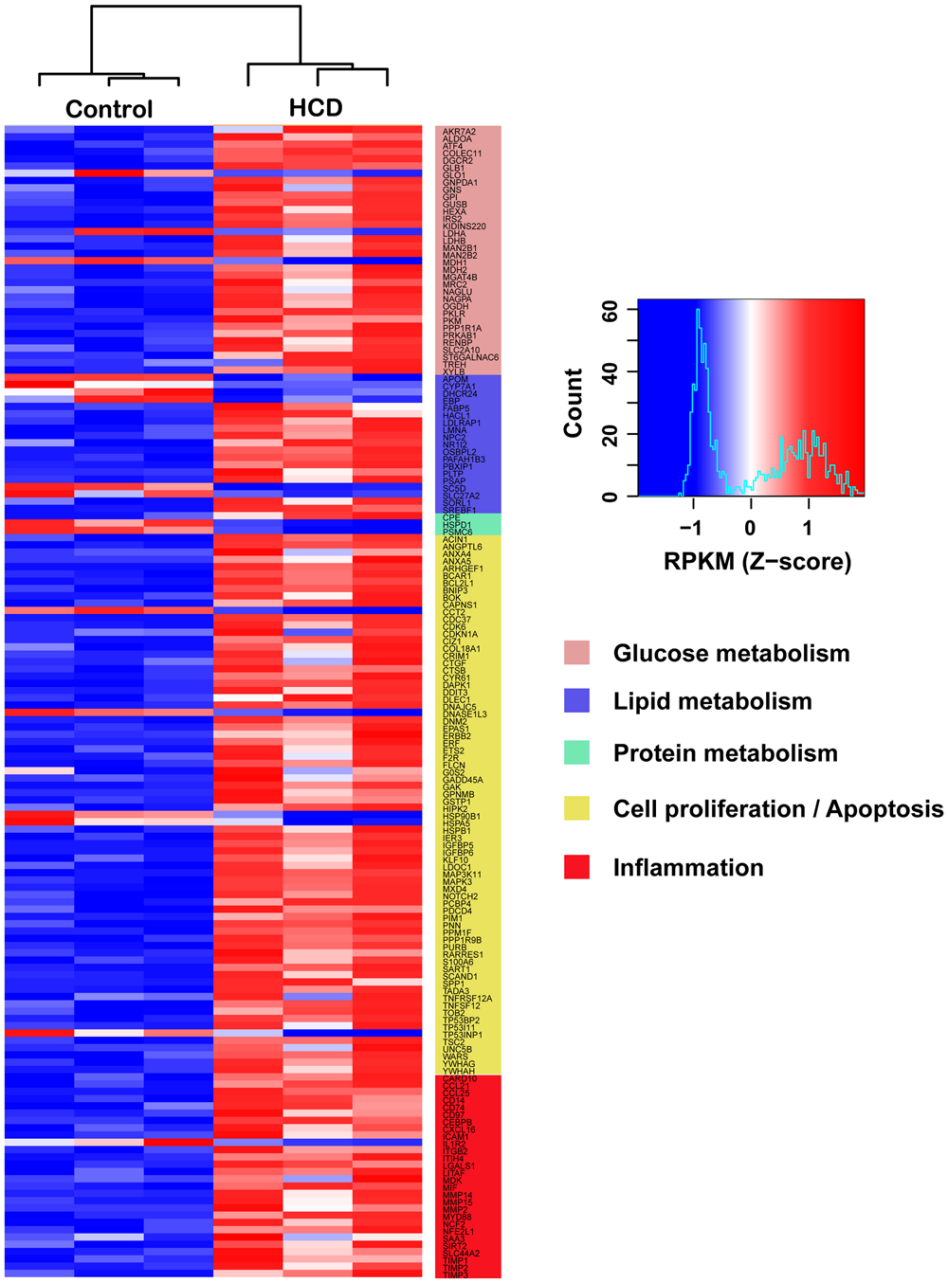
General features of differentially expressed genes in the livers of rabbits. The DEGs were analyzed using DESeq software by comparing the control and HCD groups. Expression intensities are displayed from blue (low expression) to red (high expression), and the expression level is proportional to the brightness of the color (see color bar). n = 3 for each group.

To gain insight into the complex traits underlying the pathophysiological response of the liver to dietary cholesterol, transcriptomic analysis was performed by RNA-Seq. In the previous study, we identified the gene mutations or modifiers through whole-genomic sequencing and deep transcriptome sequencing of LDL receptor deficient (WHHL) rabbits and cholesterol-fed New Zealand White rabbits. The previous study mainly addressed transcriptional changes in the WHHL rabbits, while cholesterol-fed New Zealand White rabbits were used as a reference^30^. To further emphasize the relationship with lipoprotein profiles and aortic atherosclerosis, in the current study, we focused on the hepatic gene expression changes and liver pathology in the setting of hepatic steatosis induced by cholesterol diet in the Japanese White rabbits. The transcriptomic analysis revealed that there were 1,183 differentially expressed genes (DEGs) compared with the control rabbits. Of them, 984 genes were up-regulated and 199 genes were down-regulated compared with the control group (Supplementary Table S1). Kleemann *et al*. found that 1,896 genes were significantly changed in the livers of ApoE*3Leiden mice fed a cholesterol diet during atherogenesis by GeneChip microarrays^29^. To investigate the functions of these 1,183 DEGs, we grouped them into five categories according to their functions, including glucose metabolism, lipid metabolism, protein metabolism, cell proliferation/apoptosis and inflammation. Our results revealed that HCD predominantly affected genes involved in inflammation and lipid metabolism in the liver (Fig. 5 and Supplementary Table S2). As shown in Fig. 5 and Table S1, we found that the adaptation of hepatic lipid metabolism to dietary cholesterol was mainly controlled by the genes of cholesterol biosynthesis and lipid metabolism. Sterol regulatory element binding protein 1 (SREBF1), a key transcriptional factor in modulating cholesterol biosynthesis, was up-regulated 40.7-fold compared to the control rabbits. Karasawa *et al*. revealed that hepatic SREBP1 determined plasma remnant lipoproteins and contributed to atherosclerosis in LDLR−/− mice^31^. It has been reported that 24-dehydrocholesterol reductase (DHCR24) is likely a target for SREBPs, which has been suggested to regulate the genes involved in cholesterol biosynthesis^32^. Our results showed that the DHCR24 gene was down-regulated 2.1-fold in HCD-fed rabbits. Furthermore, HCD induced many genes that mediate the inflammatory response in the liver. These genes include proteases (MMP2, MMP14, MMP15, TIMP1, TIMP2, TIMP3), chemokines (CCL25, CCL21, CXCL16, MIF), adhesion molecules (CD74, CD14, CD97, ICAM1) and cardiovascular risk factors (SAA3, CEBPB). It is generally accepted that atherosclerosis is a multifactorial and persistent condition and is regarded as a form of chronic inflammation induced by lipid accumulation^3^. Other studies showed that there is a link between cholesterol and inflammation in ApoE−/− mice and ApoE*3Leiden mice fed with high cholesterol^29,33^. Taken together, these findings suggest that hepatic lipid metabolism and inflammation may be closely associated with the initiation and progression of atherosclerosis in rabbits.

**Figure 6:**
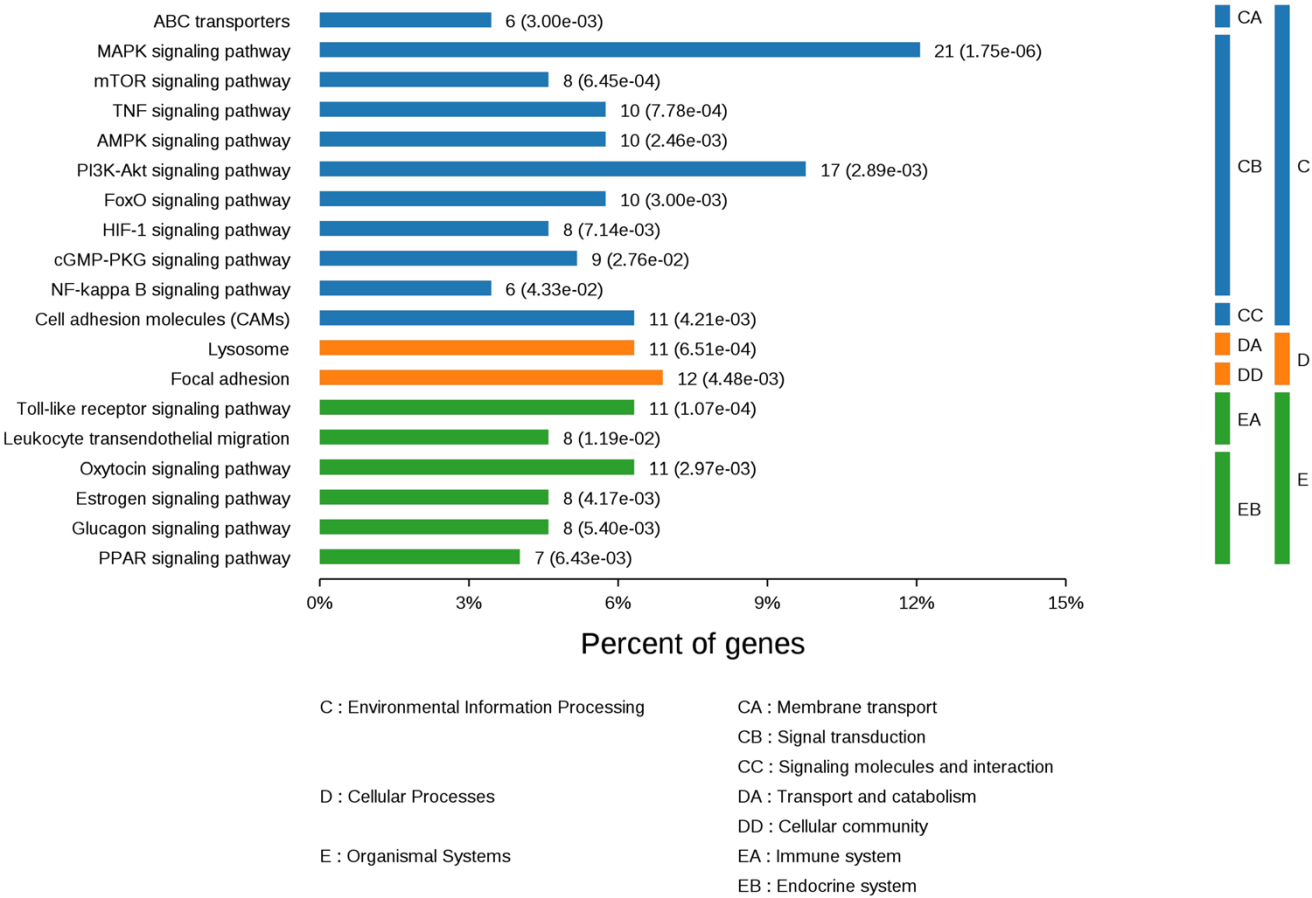
Enriched KEGG pathways of differentially expressed genes in the livers of rabbits. The DEGs were mapped into the KEGG databases, significantly enriched KEGG terms were determined by *P* ≤ 0.05. n = 3 for each group.

The Kyoto Encyclopedia of Genes and Genomes (KEGG) pathway analysis provided additional possible functional information showing the pathways relevant to DEGs during atherogenesis. In concordance with our DEG results, analysis of the KEGG pathway showed that these DEGs were mainly contained in lipid metabolism- and inflammation-related signaling pathways including PI3K-Akt, MAPK, ABC transporters, FoxO, PPAR, AMPK, NF-κB, mTOR, tumor necrosis factor (TNF), Toll-like receptor and cGMP-PKG signaling pathways. Significantly, HCD activated specific inflammatory pathways, such as NF-κB, Toll-like receptor and TNF signaling pathways in rabbits (Fig. 6 and Supplementary Table S3). As shown in Fig. 6, the number and percentage of DEGs in these signaling pathways were analyzed. We found that the largest functional pathway was the MAPK signaling pathway, representing a total of 21 DEGs (~12% of the total). It has been reported that these enriched signaling pathways participate in the inflammatory response and lipid metabolism in atherosclerosis^34,35^. Vergnes *et al*. also discovered that the hepatic inflammatory response was evoked by high cholesterol feeding in LDLR−/− mice^36^. These above studies suggest that the uptake of dietary cholesterol leads to the inflammatory response of the liver in rabbits.

### Quantitative real-time PCR analysis

**Figure 7:**
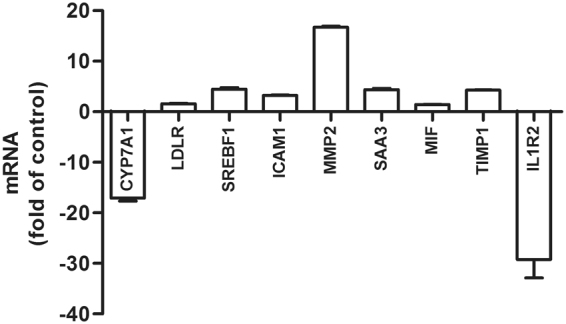
Quantitative real-time PCR verification. Real-time PCR was performed essentially to validate the DEGs in the livers of the control group and HCD group. Data are expressed as the mean ± SEM. n = 3 for each group.

Both hypercholesterolemia and inflammatory response are well-known risk factors for the development of atherosclerosis and have been shown to play a causal role in the progression of atherosclerotic plaques^3^. To validate the DEGs in the liver, real-time PCR was carried out. As shown in Fig. 7, the genes SREBF1, LDLR and CYP7A1 involved in lipid metabolism were significantly changed in the livers of the HCD group compared with the control group. Furthermore, we found that the expression levels of inflammatory genes MMP2, TIMP1, MIF, ICAM1 and SAA3 were increased, whereas the expression level of interleukin-1R2 (IL-1R2) was decreased. Previous studies showed that the proinflammatory cytokine IL-1 interacts with cells through two types of receptors, type I receptor (IL-1R1) and IL-1R2. IL-1R2 inhibits IL-1 activity by acting as a decoy target for IL-1^37,38^. These results indicate that dietary cholesterol can be an important trigger and a possible source of the inflammatory component.

**Figure 8:**
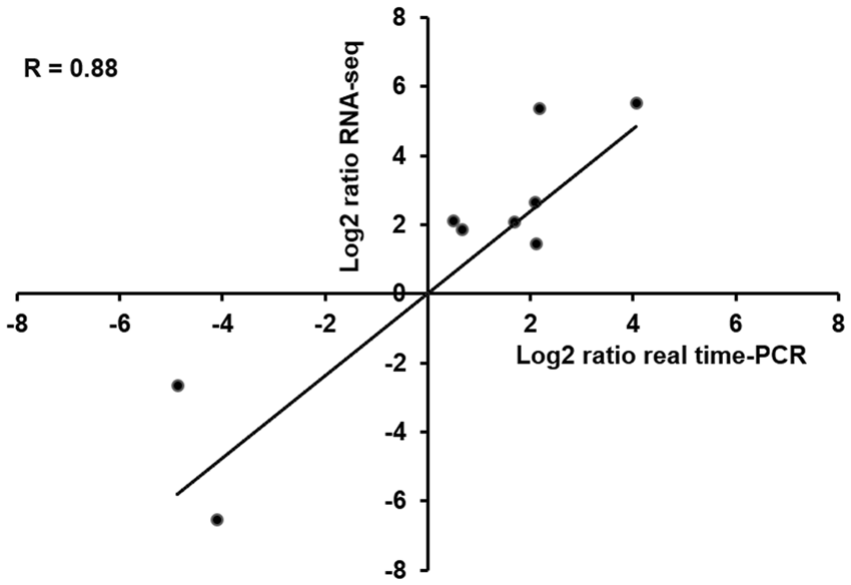
The correlation of log2-fold change in gene expression in the liver of rabbits between the RNA-Seq and real time-PCR.

The correlation was calculated to compare the expression levels between RNA-Seq and real-time PCR. Fold changes in DEGs between the two techniques were significantly correlated in the depot (Pearson’s R = 0.88) (Fig. 8). The results confirmed that these DEGs identified in our study were very reliable.

## Conclusions

The results from the current study demonstrate that dietary cholesterol is not only a risk factor for hypercholesterolemia but also a trigger of hepatic inflammation. Although the significance of hepatic inflammation in the development of atherosclerosis deserves further investigation, these results provide valuable information for the study of both hypercholesterolemia and hepatic inflammation using rabbit models. Further studies are ongoing to validate these findings.

## Materials and Methods

### Animals and diets

Male Japanese White rabbits (2.5–3.0 kg) were provided by the Laboratory Animal Center of Xi’an Jiaotong University. In the current study, we aimed to determine the influence of hypercholesterolemia on transcriptomic change and liver pathology that may be related to atherosclerosis in rabbits. It is well known that rabbits are sensitive to dietary cholesterol and rapidly develop severe hypercholesterolemia leading to atherosclerosis^5^. However, rabbits are out-bred and show a wide biological variability in terms of individual responsiveness to dietary cholesterol^39^. To minimize the intra-group variations of transcriptomic analysis caused by different response to a cholesterol diet, thirty rabbits were fed a chow diet containing 0.5% cholesterol for one week. To ensure that all rabbits had a similar responsiveness to cholesterol feeding, we selected rabbits based on the plasma levels of TC (ranging from 300 to 500 mg/dl). Eventually, twenty rabbits were selected and subsequently divided into two groups: normal chow diet group and cholesterol diet group fed a 0.3% cholesterol diet. All rabbits were given a restricted diet of 100 g/day for 16 weeks. Rabbits were individually housed in metal cages in air-conditioned rooms under a 12 h light/12 h dark cycle and were given free access to water throughout the experiments. The experimental protocol was in accordance with the National Institutes of Health Guide for Care and Use of Laboratory Animals and was approved by the Laboratory Animal Care Committee of Xi’an Jiaotong University.

### Biochemical analysis

Blood samples were collected from the ear artery of rabbits after fasting for 16 h. The plasma levels of TC and TG were measured using commercial kits (Biosino Bio-technology and Science, Beijing, China). Plasma samples were also used for lipoprotein subfraction analysis. The analysis was performed electrophoretically using high-resolution 3% polyacrylamide gel tubes and the Lipoprint LDL System (Quantimetrix Corporation, Redondo Beach, CA, USA) according to the manufacturer’s instructions as previously described^40,41^.

### Determination of atherosclerotic lesions

Rabbits were sacrificed with an injection of phenobarbital sodium and xylazine hydrochloride. The aortic tree was isolated, opened longitudinally, fixed in 10% neutral buffered formalin, and stained with Sudan IV^42^. Then, the aortic arch was cut into 10 sections. The sections were embedded in paraffin and cut into 5-μm thick serial sections, stained with H&E and EVG. For the evaluation of cellular components in atherosclerotic lesions, the sections were immunohistochemically stained with antibodies against RAM11 (macrophage marker) (Dako, Carpinteria, CA, USA) and HHF35 (SMC marker) (Thermo, Rockford, IL, USA).

### Histology and Immunohistochemistry

The livers were fixed in 10% neutral buffered formalin and processed for embedding in paraffin. Then, specimens were cut into 5-μm thick serial sections and stained with H&E. Frozen sections of liver (5-μm thick) were stained with Oil Red O for evaluation of hepatic fatty change. The Oil Red O staining area was measured using the WinROOF image analysis software (Mitani, Tokyo, Japan).

### Total RNA extraction and cDNA library construction

Total RNA was extracted from the liver using RNAzol (Takara, Tokyo, Japan). RNA concentrations and purity were determined using Nanodrop (Thermo, Rockford, IL, USA), and integrity was verified using an Agilent 2100 Bioanalyzer (Agilent Technologies, Santa Clara, CA, USA). mRNA was purified from total RNA using the NEBNext® Poly(A) mRNA magnetic isolation module. A total of 6 paired-end libraries were constructed using a NEBNext UltraTM RNA Library Prep Kit following Illumina manufacturer’s recommendation. Library quality was assessed on the Agilent Bioanalyzer 2100 system. The clustering of the index-coded samples was performed on a cBot Cluster Generation System using TruSeq PE Cluster Kit v4-cBot-HS (Illumina). The libraries were sequenced on an Illumina HiSeq. 2500 platform with 100 bp paired-end reads (Illumina, San Diego, CA, USA).

### Differentially expressed gene analysis

The Perl script was used to trim the reads with contaminated adapters, more than 20% low-quality bases (Phred quality score < 20), and more than 10% Ns. First, we obtained clean reads (Supplementary Table S4). Then, clean reads were aligned to the rabbit reference genome (GCA_000003625) using TopHat. The gene expression was quantified and normalized by Cufflinks in RPKM (reads per million per kilo bases)^43^. The DEGs were analyzed using DESeq software by comparing the control and HCD groups^44^. The significance threshold of the *p*-value in multiple tests was set by the false discovery rate (FDR). The threshold, the absolute values of fold change ≥ 2 and FDR < 0.05, was applied to judge the significance of gene expression. For the functional and pathway enrichment analysis, the DEGs were then mapped into the KEGG databases, and significantly enriched KEGG terms were determined by *P* ≤ 0.05.

### Real-time PCR

Real-time PCR was performed essentially to validate and confirm the differences in gene expression between the HCD group and the control group. Total RNA was extracted from the liver using RNAzol. cDNAs were synthesized using a RevertAid First Strand cDNA Synthesis Kit (Takara, Tokyo, Japan) following the manufacturer’s instructions. Real-time PCR was performed with the SYBR Green PCR master mix (Takara, Tokyo, Japan) using a thermal cycler dice real time system. Primers were designed with primer premier 5.0 (Premier Biosoft, Palo Alto, CA, USA) (Supplementary Table S5). GAPDH was used as an endogenous control. The relative gene expression was quantitatively analyzed by the comparative Ct method (2^−ΔΔCT^). Data were normalized to rabbit GAPDH mRNA levels.

### Statistical analysis

Data are presented as the mean ± SEM. Statistical analysis was performed by the two-tailed Student’s t-test using GraphPad Prism software. Differences between groups were considered statistically significant if *P* < 0.05.

## Electronic supplementary material

Table S1

Table S2

Table S3

Table S4

Table S5
