# Supplementary material for: Transcriptomic analysis of the liver of cholesterol-fed rabbits reveals altered hepatic lipid metabolism and inflammatory response
Source: Sci Rep. 2018 Apr 24;8:6437. doi: 10.1038/s41598-018-24813-1 (PMC5915436; doi:10.1038/s41598-018-24813-1)
Supplement: Supplementary file 2 — Table S2 [file 41598_2018_24813_MOESM2_ESM.doc]

**Table S2**

Overview of hepatic differentially expressed genes in response to cholesterol in rabbits

| Gene category | HCD | | |
| --- | --- | --- | --- |
|  | Up | Down | Total |
| Inflammation | 27 | 1 | 28 |
| Lipid metabolism | 13 | 6 | 19 |
| Cell Proliferation/Apoptosis | 69 | 5 | 74 |
| Glucose metabolism | 31 | 3 | 34 |
| Protein metabolism | 1 | 2 | 3 |

Differentially expressed genes between HCD and control group were analyzed according to the functional categories.
