# Supplementary material for: Transcriptomic analysis of the liver of cholesterol-fed rabbits reveals altered hepatic lipid metabolism and inflammatory response
Source: Sci Rep. 2018 Apr 24;8:6437. doi: 10.1038/s41598-018-24813-1 (PMC5915436; doi:10.1038/s41598-018-24813-1)
Supplement: Supplementary file 4 — Table S4 [file 41598_2018_24813_MOESM4_ESM.doc]

**Table S4**

Summary of the RNA-seq data in the rabbits with high cholesterol diet

| Group | Clean Reads | Total Sequence | Insert  Size (bp) | Sequence Length | GC (%) | Q20 (%) | Q30 (%) |
| --- | --- | --- | --- | --- | --- | --- | --- |
| Control-1 | 44,374,856 | 4,432,760,022 | 250 | 100 | 52 | 98.61 | 93.23 |
| Control-2 | 50,882,722 | 5,082,247,947 | 250 | 100 | 52 | 98.67 | 93.46 |
| Control-3 | 42,685,392 | 4,262,771,110 | 250 | 100 | 52 | 98.67 | 93.46 |
| HCD-1 | 72,518,470 | 7,193,315,426 | 250 | 100 | 57 | 98.91 | 97.79 |
| HCD-2 | 77,392,174 | 7,678,565,885 | 250 | 100 | 55 | 98.91 | 97.80 |
| HCD-3 | 55,690,768 | 5,514,503,586 | 250 | 100 | 55 | 98.83 | 97.71 |
