# Supplementary material for: Transcriptomic analysis of the liver of cholesterol-fed rabbits reveals altered hepatic lipid metabolism and inflammatory response
Source: Sci Rep. 2018 Apr 24;8:6437. doi: 10.1038/s41598-018-24813-1 (PMC5915436; doi:10.1038/s41598-018-24813-1)
Supplement: Supplementary file 5 — Table S5 [file 41598_2018_24813_MOESM5_ESM.doc]

**Table S5**

Primer sequence used for real-time PCR analysis

| Gene | Primer | Sequence (5’-3’) |
| --- | --- | --- |
| MMP-2 | Forward  Reverse | 5’- AGGACTACGACCGCGACAA-3’  5’- AGGAAGGTGAAGGGGAAGACA-3’ |
| TIMP-1 | Forward  Reverse | 5’-CTGAAGGCTGCTCCTGTTGG-3’  5’-TGTGTGGGACAAAGAAAGATGG-3’ |
| MIF | Forward  Reverse | 5’-ACCAGATCATGGCGTTCG-3’  5’-CTTGCTGTAGGTGCGGTTCT-3’ |
| ICAM-1 | Forward  Reverse | 5’-ACAGCCCGCACAGACACTT-3’  5’-CCCTGTCCACCACTCCATTC-3’ |
| SAA3  SREBF1  LDLR  CYP7A1  IL-1R2 | Forward  Reverse  Forward  Reverse  Forward  Reverse  Forward  Reverse  Forward  Reverse | 5’-GCAACGACCCCAATCACTTC-3’  5’-TGTCCCAGCAGGCTCAGTT-3’  5'-GCAGGGTGACAGGACACAAG-3'  5'-AGACCAAGGAAGGAGGGACAA-3'  5’-GGCAGCTACAAGTGCGAGTG-3’  5’-CGGTTGGTGAAGAAGAGGTAGG-3’  5’-TCAGAGACACCCTTGCCTTTC-3’  5’-AGTCCCAGAATAAGCCACAAACA-3’  5’-AGGAAATCCCAGCTCCCAAGA-3’  5’-CTCCTGACAACTTCCAGAGGACAC-3’ |
| GAPDH | Forward  Reverse | 5’-GTGATGCTGGTGCCGAGTA-3’  5’-AGGATGCGTTGCTGACAATC-3’ |
